# General versus regional anaesthesia for caesarean section indicated for acute foetal distress: a retrospective cohort study

**DOI:** 10.1186/s12871-021-01289-7

**Published:** 2021-03-04

**Authors:** Junette Arlette Mbengono Metogo, Theophile Njamen Nana, Brian Ajong Ngongheh, Emelinda Berinyuy Nyuydzefon, Christoph Akazong Adjahoung, Joel Noutakdie Tochie, Jacqueline Ze Minkande

**Affiliations:** 1 Department of Anaesthesiology and Critical Care, Douala General Hospital, Douala, Cameroon; 2 Department of Surgery and Specialties, Faculty of Medicine and Pharmaceutical Sciences, University of Douala, 1 Yaoundé, Douala, Cameroon; 3 Department of Obstetrics and Gynaecology, Douala General Hospital, Douala, Cameroon; 4 Department of Obstetrics and Gynaecology, Faculty of Health Sciences, University of Buea, Buea, Cameroon; 5 Migration Health Department, International Organization for Migration (IOM) Country Program, Kinshasa, Democratic Republic of Congo; 6 Department of Surgery and Specialties, Faculty of Medicine and Biomedical Sciences, University of Yaoundé 1, Yaoundé, Cameroon; 7 Department of Paediatrics, Douala General Hospital, Douala, Cameroon; 8 Department of Paediatrics, Faculty of Medicine and Pharmaceutical Sciences, University of Douala, Douala, Cameroon; 9 Department of Anaesthesiology and Critical Care, Yaoundé Gynaeco-Obstetric and Paediatric Hospital, Yaoundé, Cameroon

**Keywords:** Acute foetal distress, Caesarean section, Anaesthesia, Neonatal, Maternal, Outcome

## Abstract

**Background:**

Acute foetal distress (AFD) is a life-threatening foetal condition complicating 2% of all pregnancies and accounting for 8.9% of caesarean sections (CS) especially in developing nations. Despite the severity of the problem, no evidence exists as to the safest anaesthetic technique for the mother and foetus couple undergoing CS for AFD. We aimed to compare general anaesthesia (GA) versus regional (spinal and epidural) anaesthesia in terms of their perioperative maternal and foetal outcomes.

**Methods:**

We carried out a retrospective cohort study by reviewing the medical records of all women who underwent CS indicated for AFD between 2015 to 2018 at the Douala General Hospital, Cameroon. Medical records of neonates were also reviewed. We sought to investigate the association between GA, and regional anaesthesia administered during CS for AFD and foetal and maternal outcomes. The threshold of statistical significance was set at 0.05.

**Results:**

We enrolled the medical records of 117 pregnant women who underwent CS indicated for AFD. Their mean age and mean gestational age were 30.5 ± 4.8 years and 40 weeks respectively. Eighty-three (70.9%), 29 (24.8%) and 05 (4.3%) pregnant women underwent CS under SA, GA and EA respectively. Neonates delivered by CS under GA were more likely to have a significantly low APGAR score at both the 1st (RR = 1.93, *p* = 0.014) and third-minute (RR = 2.52, *p* = 0.012) and to be resuscitated at birth (RR = 2.15, *p* = 0.015). Past CS, FHR pattern on CTG didn’t affect these results in multivariate analysis. Adverse maternal outcomes are shown to be higher following SA when compared to GA.

**Conclusion:**

The study infers an association between CS performed for AFD under GA and foetal morbidity. This, however, failed to translate into a difference in perinatal mortality when comparing GA vs RA. This finding does not discount the role of GA, but we emphasize the need for specific precautions like adequate anticipation for neonatal resuscitation to reduce neonatal complications associated with CS performed for AFD under GA.

## Background

Acute foetal distress (AFD) is defined as sudden lack or shortage of oxygen supply via the bloodstream to the foetus, leading to foetal hypoxia, hypercapnia and metabolic acidosis [1, 2]. It is a non-specific term which engulfs abnormal clinical findings suggestive of a compromised foetal wellbeing most often seen during the intra-partum period. AFD clinically manifests with an abnormal foetal heart rate (tachycardia ≥160 beats/minutes or bradycardia ≤110 beats/minutes) on cardiotocography (CTG) [2, 3], intermittent doppler or hand-held fetoscope auscultation. It is a foetal and obstetrical emergency, complicating up to 2% [1] of all pregnancies and accounting for 8.9% of births due to caesarean section (CS) especially in developing countries [4]. Mortality still remains high at 31.25 per 1000 deliveries, especially when the interval between the start of AFD and childbirth exceeds 30 min [1, 4]. The management of AFD requires the safest and fastest possible route of delivery to prevent avert perinatal morbidity and mortality compounded after delivery by neonatal asphyxia (NA) or hypoxic ischemic encephalopathy [5].

However, there is an on-going debate on the safest anaesthetic technique for CS indicated for AFD in anaesthesiology. This is more challenging in low-income countries (LICs), due to insufficient health infrastructure, unfunded and unstaffed healthcare systems [6]. Comparing regional (spinal and epidural) to general techniques of anaesthesia, although general anaesthesia (GA) has the merit of rapidity in induction of anaesthesia which is invaluable for this obstetrical emergency, (AFD), when compared to regional anaesthesia techniques (RATs) [7, 8] GA could have deleterious and potentially lethal consequences to neonates born through CS performed under GA. This is due to the trans-placental passage of hypnotics, and opioids derivatives which can lead to depression of cardiovascular, respiratory and neurological functions of the neonate [9]. This later could be associated with neonatal resuscitation intensive care unit admission and nosocomial infections in the neonate. RATs, though associated with better APGAR scores both in the 1st and 5th minutes following delivery take a longer time to be realized, a factor non-desirable in women undergoing CS indicated for AFD [10]. This delay in induction time for a foetus in acute distress may worsen the consequences of AFD such as hypoxic ischemic encephalopathy, cerebral palsy, neonatal anaemia, metabolic acidosis in the new-born and ultimately, death. However, studies are yet to prove this hypothesis if true. Also, RATs have been shown to be associated with less maternal blood loss and greater postoperative satisfaction in elective CS, but not in the case of AFD [8]. A systematic review of 22 randomized and quasi-randomized controlled studies by the Cochrane Group assessed the effects of RATs versus GA on maternal and neonatal outcomes following CS [8]. However, most studies included in the review involved elective CS with only two studies involving emergency CS for AFD. Their findings, therefore, cannot be readily applied to women undergoing anaesthesia for emergency CS in AFD. There is, therefore, a need for more studies comparing both RAT and GA for CS indicated for AFD to generate more evidence required for decision making in daily clinical practice.

Against this background, we sought to compare the maternal and neonatal outcomes of women undergoing emergency CS for AFD under either RATs or GA. The research goal was to contribute to providing information necessary, to generate evidence for the safest anaesthetic technique in the case of this emergency.

## Methods

### Study design and setting

The study was carried out and reported in accordance to the STROBE checklist for cohort studies. This was a retrospective cohort study conducted at the Douala General Hospital (DGH), Douala, Cameroon. Douala is an urban zone and the economical capital of Cameroon with a population 3 million inhabitants [11]. The DGH is a university teaching hospital and one of the major reference hospitals in Cameroon. It has an Obstetrics and Gynaecology department, a Neonatal unit and an Anaesthesiology and Intensive Care department. Its neonatal unit serves as the main referral neonatal unit for all health centres in Douala and its environs. Every year, an average of 1100 childbirths occur in its Obstetrics and Gynaecology department [12] some within the delivery room (in case of vaginal delivery) and others within the operating room of the department of Anaesthesiology and Intensive Care.

### Sampling and study participants

A chart review of the obstetrical and anaesthesiology records was conducted for all consecutive parturients who underwent an emergency CS indicated for AFD between January 01, 2015, to August 31, 2018. We also reviewed the records of the neonates of these women who delivered via emergency CS. We excluded parturients with contraindications for regional anaesthesia (coagulation abnormalities, infection at the point of lumbar puncture, and haemodynamic instability, pre-existing neurological pathologies such as epilepsy, space occupying lesions or radiculopathies) as well as cases of extreme emergencies such as cord prolapse.

### Variables studied

Data extracted from the obstetrical and anaesthesiology records of parturients and neonates included maternal age, past history (parity, gravidity, comorbidities and previous CS), the anaesthesia technique whether regional (spinal or epidural) or general. Parameters specific to anaesthesiology consultation included ASA score and intubability criteria namely the mallampati score. Information on perioperative complications (immediate and late) such as post-partum haemorrhage (PPH), post-dural puncture headache (PDPH), post-operative wound infection and duration of hospitalisation were also collected. Pathologic cardiotocographic (CTG) tracings were used to group patients into two groups of foetal heart rate pattern; non-reassuring and abnormal group, according to the NICE guidelines on intra-partum care [13] Those with at least 2 non reassuring features were under the ‘non-reassuring group’ and those with an abnormal feature under the “abnormal group.” Neonatal parameters collected from hospital files of new-borns included gestational age, birth weight, APGAR scores at 1st, 3rd and 5th minutes after delivery, neonatal resuscitation and clinical diagnosis of neonatal asphyxia according to the Sarnat classification [14]. Hospitalization in the neonatal unit as well as its duration was noted. Other maternal parameters included maternal age, gestational age, and referral or not to the DGH. Maternal hypotension was defined as a drop between the pre-anaesthesia and post-induction of anaesthesia systolic blood pressure by 30%.

### Data analysis

Data was extracted from the medical records of the participants into a structured data collection sheet. The data was entered into Epi-info version 7.1.3.3 software. The primary exposure of interest was the type of anaesthesia administered, whether regional (spinal, epidural) or general anaesthesia (GA). The primary outcomes of interest were the foetal outcomes after delivery which included: APGAR score at the 1st, 3rd and 5th minutes, a clinical diagnosis of neonatal asphyxia according to the Sarnat classification [12], neonatal resuscitation. We conducted univariable analysis followed by a multivariable analysis to adjust for possible confounders. Risk ratios (RR) with their corresponding 95% confidence intervals (95% CI) and *p*-values were calculated to measure associations. Variables with missing data precluding meaningful analyses were excluded. A *p*-value < 0.05 was considered statistically significant.

## Results

### Socio-demographic characteristics of parturients

A total of 117 women met the eligibility criteria and were included into the study. The mean age was 30.5 ± 4.8 years (range: 18 to 42 years). Among the women included in the study, 18 (15.4%) were referred from other health facilities to DGH for CS delivery. The main reasons for referral included: stationary labour, post-term delivery, acute foetal distress (AFD), pre-eclampsia and preterm labour. The gestational age of pregnancy ranged from 30 to 42 weeks, with a median gestational age of 40 weeks (IQR: 39–40 weeks). The gravidity of participants ranged from 1 to 8 with a median gravidity of 2 (IQR = 1 to 4). Few women, 16 (13.68%) had undergone a previous CS.

### Neonatal characteristics

Most of the babies born to the participants were males (61.11%). All the neonates were born alive. The APGAR score at birth ranged from 2 to 10, with a median of 8 (IQR 6 to 8). Table 1 summarizes the APGAR scores of new-borns at birth and after the 3rd and 5th minutes of life.

**Table 1:** Characteristics of the APGAR score of neonates at birth, 3 and 5 min of life

APGAR	Range	Median	IQR
At birth	2 to 10	8	6 to 8
At 3 min	3 to 10	9	7 to 10
At 5 min	5 to 10	10	8 to 10

Amongst the new-borns, 42 were diagnosed of neonatal asphyxia giving a cumulative incidence of 35.9%. About two thirds (64.3%) of the new-borns with neonatal asphyxia were classified as Sarnat class 1, meanwhile 21.4 and 14.3% were Sarnat class 2 and 3 respectively. The duration of hospitalization of the new-borns in the neonatal unit ranged from 2 to 43 days with a median of 4 days (IQR: 2 to 21 days). A total of 29 neonates (24.8%) were resuscitated at birth. Table 2 summarizes the characteristics of the new-borns.

**Table 2:** Summary of new-born characteristics

Variable	Category	Number (%)	Percentage (%)
**Neonatal asphyxia**	Yes	42	35.9
	No	75	64.1
**Sarnat classification**	Sarnat 1	27	64.3
	Sarnat 2	9	21.4
	Sarnat 3	6	14.3
**Birth Weight**	Low	16	15.4
	Normal	88	84.6
**Immediate cry**	Yes	27	23.1
	No	90	76.9
**Resuscitation**	No	88	75.2
	Yes	29	24.8
**Apgar at birth**	Low	36	30.8
	Normal	81	69.2
**Apgar at 3 min**	Low	22	18.8
	Normal	95	81.2
**Apgar at 5 min**	Low	8	6.8
	Normal	109	93.2
**Sex**	Male	66	61.1
	Female	42	38.9

### Clinical parameters of parturients

On admission, most of the parturients had a good general state 112 (95.7%). The ASA score was II in 75 (64.10%), III in 38 (32.48%) and IV in 4 (3.42%) of the parturients. Fever was present in 18 (15.4%) of participants.

### Type of anaesthetic technique used for emergency caesarean section indicated for AFD

More than two thirds 83 (70.9%) of the participants underwent CS for AFD under spinal anaesthesia (SA); 29 (24.8%) under general anaesthesia; and five (4.3%) under epidural anaesthesia (EA). Parturients who underwent emergency CS for AFD under epidural anaesthesia had the epidural catheter placed before delivery for obstetrical analgesia. This was converted to obstetrical anaesthesia immediately within the operating room when CS was indicated for AFD. Spinal anaesthesia was administered with the patient in the sitting position. Among patients administered GA, the most used hypnotic for induction was propofol 24 (82.8%). Thiopental and ketamine were used in 3 and 2 parturients, respectively. Bupivacaine (Bupi) with fentanyl as adjuvant was used in 79 (95.2%) patients operated under SA. Bupivacaine only and combinations of Bupi+morphine or Bupi+morphine+Fentanyl were used in two, one and one case, respectively. Bupivacaine was used for epidural anaesthesia. Hypotension during regional anaesthesia was managed with the use of ephedrine in 32 (27.3%) of parturients. The mean interval from induction of anaesthesia to extraction of the foetus in women administered SA (22.4 ± 9.9 min) was significantly longer compared to the interval in women administered GA (8.0 ± 4.9 min) (*p* <  0.001).

### Maternal outcome after surgery

Immediate postoperative complications were few. In the GA group, we had one case of Post-partum haemorrhage (PPH). In the SA group, there were 2 cases of PPH, one of the cases being complicated by maternal death. Furthermore, one case of severe hypotension was noted. No maternal complications were noted in the epidural anaesthesia group. Duration of hospitalisation of mothers ranged from 3 to 13 days with a mean of 5 days. Mean duration of hospitalization for women who underwent CS under SA was 4.5 days and 4.7 days for GA (*p* = 0.5154). Late complications observed during hospitalization were seen mostly in postpartum women who underwent SA. These included four cases of post-dural puncture headaches, two cases of endometritis, one case of malaria and one case surgical site infection.

### Univariable analysis

#### Association of predictor variables with APGAR at the first minute

In univariable analysis, babies born of women who received GA had two-fold increase risk of having a low APGAR score at the first minute compared to babies of women who received SA (RR = 2.00, *p* = 0.011). Furthermore, neonates born of women with fever almost had a double likelihood of having a low APGAR score at the first minute compared to babies born of women without fever (RR = 1.83, *p* = 0.035). On the other hand, gravidity, FHR pattern, use of epinephrine, gestational age, birth weight and previous CS were not associated with low APGAR in univariable analysis. Table 3 shows the association between the exposure variables and APGAR at the first minute in univariable analysis.

**Table 3:** Association between exposure variables with APGAR at first minute

Exposure	Categories	APGAR at birth				
		≥ 7 (%)	< 7 (%)	RR	***p*** value	95% CI
**Type of Anaesthesia administered**	Spinal	63 (80.7)	20 (58.8)	1	–	–
	General	15 (19.2)	14 (41.2)	2.00	0.011	1.17–3.42
**Gravidity**	Multi-Gravida	60 (74.1)	26 (72.2)	1	–	–
	Primi-gravida	21 (25.9)	10 (27.8)	1.06	0.833	0.58–1.95
**Type of FHR**	Abnormal	70 (86.4)	29 (80.6)	1	–	–
	Non-Reassuring	11 (13.6)	7 (19.4)	1.32	0.396	0.69–2.55
**Referral**	Not referred	69 (85.2)	30 (83.3)	1	–	–
	Referred	12 (14.8)	6 (16.7)	1.10	0.795	0.53–2.26
**Use of Ephedrine**	No	57 (70.4)	28 (77.8)	1	–	–
	Yes	24 (29.6)	8 (22.2)	0.76	0.421	0.39–1.49
**Gestational age**	Term	81 (63.0)	21 (58.3)	1	–	–
	Premature	6 (7.4)	7 (19.5)	1.85	0.052	0.99–3.43
	Post term	24 (29.6)	8 (22.2)	0.86	0.666	0.42–1.72
**Birth Weight**	Normal or high	65 (86.7)	23 (79.3)	1	–	–
	Low	10 (13.3)	6 (20.7)	1.43	0.328	0.69–2.96
**Previous CS**	No	66 (81.5)	35 (97.2)	1	–	–
	Yes	15 (18.5)	1 (2.8)	0.18	0.080	0.03–1.22
**Fever**	No	72 (88.9)	27 (75.0)	1	–	–
	Yes	9 (11.1)	9 (25.0)	1.83	0.035	1.04–3.22

#### Association of exposure variables with APGAR at the third minute

Babies born of women who underwent GA had almost three times the risk of having a low APGAR score at the third minute compared to babies of women who underwent SA (RR = 2.86, *p* = 0.007). Furthermore, neonates delivered by women with fever had three times the risk of having a low APGAR score at the third minute compared to babies born of women without fever (RR = 3.14, *p* = 0.002). On the other hand, gravidity, FHR pattern, use of epinephrine, gestational age, birth weight and previous CS were not associated with a low APGAR score at the third minute. Table 4 shows the association between the exposure variables and APGAR score at the third minute.

**Table 4:** Association between exposure variables and APGAR at third minute

Exposure	Category	APGAR 3rd minute				
		≥ 7 (%)	< 7 (%)	RR	***p*** value	95% CI
**Type of Anaesthesia administered**	Spinal	73 (79.3)	10 (50.0)	1	–	–
	General	19 (20.6)	10 (50.0)	2.86	0.007	1.33–6.17
**Gravidity**	Multi-Gravida	70 (73.7)	16 (72.7)	1	–	–
	Primi-gravida	25 (26.3)	6 (27.3)	1.04	0.927	0.45–2.42
**Type of FHR**	Abnormal	80 (84.2)	19 (86.4)	1	–	–
	Non-Reassuring	15 (15.8)	3 (13.6)	0.87	0.803	0.29–2.63
**Referral**	Not referred	80 (84.2)	19 (86.4)	1	–	–
	Referred	15 (15.8)	3 (13.6)	0.87	0.803	0.28–2.63
**Use of Ephedrine**	No	65 (68.4)	20 (90.9)	1	–	–
	Yes	30 (31.6)	2 (9.1)	0.27	0.063	0.06–1.07
**Gestational age**	Term	59 (62.1)	13 (59.1)	1	–	–
	Premature	10 (10.5)	3 (13.6)	1.28	0.664	0.42–3.87
	Post term	26 (27.4)	6 (27.3)	1.03	0.932	0.43–2.49
**Birth Weight**	Normal or high	74 (87.1)	14 (73.7)	1	–	–
	Low	11 (12.9)	5 (26.3)	1.96	0.129	0.82–4.69
**Previous CS**	No	80 (84.2)	21 (95.6)	1	–	–
	Yes	15 (15.8)	1 (4.6)	0.30	0.224	0.04–2.08
**Fever**	No	85 (89.5)	14 (63.6)	1	–	–
	Yes	10 (10.5)	8 (36.4)	3.14	0.002	1.54–6.38

### Association of exposure variables with APGAR at the fifth minute

The type of anaesthesia administered, maternal fever, gravidity, the type of FHR, use of epinephrine, gestational age, birth weight and previous CS were not significantly associated with low APGAR at the fifth minute. Table 5 shows the association between the exposure variables and APGAR at the fifth minute.

**Table 5:** Association between exposure variables with APGAR at fifth minute

Exposure	Category	APGAR at 5th minute				
		≥ 7(%)	< 7(%)	RR	***p*** value	95% CI
**Type of Anaesthesia administered**	Spinal	79 (76.0)	4 (50.0)	1	–	–
	General	25 (24.0)	4 (50.0)	2.86	0.118	0.76–10.71
**Gravidity**	Multi-Gravida	82 (75.2)	4 (50.0)	1	–	–
	Primi-gravida	27 (24.8)	4 (50.0)	2.77	0.131	0.74–10.42
**Type of FHR**	Abnormal	92 (84.4)	7 (87.5)	1	–	–
	Non-Reassuring	17 (15.6)	1 (12.5)	0.79	0.816	0.10–6.01
**Referral**	Not referred	93 (85.3)	6 (75.0)	1	–	–
	Referred	16 (14.7)	2 (25.0)	1.83	0.434	0.40–8.38
**Use of Ephedrine**	No	79 (72.5)	6 (75.0)	1	–	–
	Yes	30 (27.5)	2 (25.0)	0.88	0.878	0.19–4.16
**Gestational age**	Term	66 (60.6)	6 (75.0)	1	–	–
	Premature	13 (11.9)	0 (0.00)	–	–	–
	Post term	30 (27.5)	2 (25.0)	0.75	0.715	0.16–3.52
**Birth Weight**	Normal or high	83 (84.7)	5 (83.3)	1	–	–
	Low	15 (15.3)	1 (16.7)	1.1	0.928	0.14–8.80
**Previous CS**	No	93 (85.3)	8 (100.0)	1	–	–
	Yes	16 (14.7)	0 (0.0)	–	–	–
**Fever**	No	93 (85.3)	6 (75.0)	1	–	–
	Yes	16 (14.7)	2 (25.0)	1.83	0.434	0.40–8.38

### Association of exposure variables and neonatal resuscitation

New-borns of women who underwent GA were twice at risk of being resuscitated compared to those born of women who received spinal anaesthesia (*P* = 0.015). Table 6 shows the association between the exposure variables and neonatal resuscitation.

**Table 6:** Association between exposure variables and neonatal resuscitation

Exposure	Category	Resuscitation				
		Yes (%)	No (%)	RR	***p*** value	95% CI
**Type of Anaesthesia administered**	Spinal	16 (57.1)	67 (79.8)	1	–	–
	General	12 (42.9)	17 (20.2)	2.15	0.015	1.16–3.98
**Gravidity**	Multi-Gravida	21 (72.4)	65 (73.9)	1	–	
	Primi-gravida	8 (27.6)	23 (26.1)	1.06	0.878	0.52–2.13
**Type of FHR**	Abnormal	25 (86.2)	74 (84.1)	1	–	–
	Non-Reassuring	4 (13.8)	14 (15.9)	0.88	0.787	0.35–2.23
**Referral**	Not referred	28 (96.6)	71 (80.7)	1	–	–
	Referred	1 (3.4)	17 (19.3)	0.19	0.98	0.03–1.35
**Use of Ephedrine**	No	23 (79.3)	62 (70.5)	1	–	–
	Yes	6 (20.)	26 (29.5)	0.69	0.370	0.31–1.54
**Gestational age**	Term	20 (69.0)	52 (59.1)	1	–	–
	Premature	2 (6.9)	11 (12.5)	0.55	0.383	0.15–2.09
	Post term	7 (24.1)	25 (28.4)	0.79	0.534	0.37–1.67
**Birth Weight**	Normal or high	20 (83.3)	68 (85.0)	1	–	–
	Low	4 (16.7)	12 (15)	1.1	0.841	0.43–2.79
**Previous CS**	No	26 (89.7)	75 (85.2)	1	–	–
	Yes	3 (10.3)	13 (14.8)	0.73	0.562	0.25–2.13
**Fever**	No	23 (79.3)	76 (86.4)	1	–	–
	Yes	6 (20.7)	12 (13.6)	1.43	0.342	0.68–3.02

### Association of exposure variables with neonatal asphyxia (NNA)

In univariable analysis, the type of anaesthesia administered was not significantly associated with the clinical diagnosis of NNA. Furthermore, gravidity, the type of FHR pattern, gestational age, birth weight, previous CS, the use of epinephrine and the presence of postoperative fever were not associated with NNA in univariable analysis. Table 7 shows the association between the exposure variables and NNA.

**Table 7:** Association between exposure variables with NNA

Exposure	Category	Neonatal Asphyxia				
		No (%)	Yes (%)	RR	***P*** value	95% CI
**Type of Anaesthesia administered**	Spinal	54 (75.0)	29 (72.5)	1	–	–
	General	17 (22.7)	11 (22.5)	1.08	0.770	0.63–1.88
**Gravidity**	Multi-Gravida	58 (77.3)	28 (66.7)	1	–	–
	Primi-gravida	17 (22.7)	14 (33.3)	1.39	0.193	0.85–2.27
**Type of FHR pattern**	Abnormal	65 (86.7)	34 (80.9)	1	–	–
	Non-Reassuring	10 (13.3)	8 (19.1)	1.29	0.387	0.72–2.32
**Referral**	Not referred	65 (86.7)	34 (80.9)	1	–	–
	Referred	10 13.3)	8 (19.1)	1.29	0.387	0.72–2.32
**Use of Ephedrine**	No	53 (70.7)	32 (76.2)	1	–	–
	Yes	22 (29.3)	10 (23.8)	0.83	0.531	0.46–1.48
**Gestational age**	Term	42 (56.0)	30 (71.4)	1	–	–
	Premature	10 (13.3)	3 (7.2)	0.55	0.261	0.20–1.55
	Post term	23 (30.7)	9 (21.4)	0.67	0.212	0.36–1.25
**Birth Weight**	Normal or high	57 (81.4)	31 (91.2)	1	–	
	Low	13 (18.6)	3 (8.8)	0.53	0.243	0.18–1.53
**Previous CS**	No	63 (84.0)	38 (90.5)	1	–	–
	Yes	12 (16.0)	4 (9.5)	0.66	0.365	0.27–1.61
**Fever**	No	65 (86.7)	34 (80.9)	1	–	–
	Yes	10 (13.3)	8 (19.1)	1.29	0.387	0.72–2.32

### Association of exposure variables and new-born hospitalization in the neonatal unit

There was no significant difference in the chances of neonates being hospitalised in the neonatal unit for both SA and GA. However, post term new-borns were about 50% less likely to be hospitalized in the neonatal unit compared to neonates born at term(*P* = 0.035). Furthermore, babies with low birth weights were about twice as likely to be hospitalised (*P* = 0.001). Table 8 shows the association of exposure variables with hospitalization of new-borns in the neonatal unit.

**Table 8:** Association between exposure variables and neonatal unit admission

Exposure	Category	Hospitalization in the neonatal unit				
		Yes (%)	No (%)	RR	***p*** value	95% CI
**Type of Anaesthesia administered**	Spinal	43 (70.5)	40 (78.4)	1	–	–
	General	18 (29.5)	11 (21.6)	1.20	0.314	0.84–1.70
**Gravidity**	Multi-Gravida	44 (68.8)	42 (79.3)	1	–	
	Primi-gravida	20 (31.2)	11 (20.7)	1.26	0.172	0.90–1.76
**Type of FHR**	Abnormal	53 (82.8)	46 (86.8)	1	–	–
	Non-Reassuring	11 (17.2)	7 (13.2)	1.14	0.529	0.76–1.72
**Referral**	Not referred	52 (81.2)	47 (88.7)	1	–	–
	Referred	12 (18.8)	6 (11.3)	1.27	0.215	0.87–1.85
**Use of Epinephrine**	No	49 (76.6)	36 (67.9)	1	–	–
	Yes	15 (23.4)	17 (32.1)	0.81	0.324	0.54–1.23
**Gestational age**	Term	43 (67.2)	29 (54.7)	1	–	–
	Premature	10 (15.6)	3 (5.7)	1.29	0.160	0.90–1.83
	Post term	11 (17.2)	21 (39.6)	0.57	0.035	0.34–0.96
**Birth Weight**	Normal or high	40 (74.1)	48 (96.0)	1	–	–
	Low	14 (25.9)	2 (4.0)	1.92	< 0.001	1.43–2.58
**Previous CS**	No	57 (89.1)	44 (83.0)	1	–	–
	Yes	7 (10.9)	9 (17.0)	0.77	0.391	0.43–1.39
**Fever**	No	53 (82.8)	46 (86.9)	1	–	–
	Yes	11 (17.2)	7 (13.2)	1.14	0.529	0.76–1.72

### Association of exposure variables and duration of neonatal unit hospitalization

Neonates born following GA were likely to have a longer mean duration of hospitalisation in the neonatal unit compared to those born under SA 4.7 days versus 4.5 days. However, the difference in mean was not statistically significant (p = 0.515).

### Multivariable analysis

After accounting for foetal heart rate patterns on CTG, past caesarean scar and whether parturients were referred from another hospital, SA remained significantly associated with better APGAR scores in the first and 3rd minute following Caesarean section, and with lesser risks of resuscitation of new-borns (*P* < 0.05). Table 9 shows the association between anaesthesia technic and main outcome variables in multivariable analysis.

**Table 9:** Association between anaesthesia technique and outcome variables in multivariate analysis

Exposure		ARR	CI	***P***-Value
**APGAR at birth**				
**Type of anaesthesia**	Regional	1	–	0.016
	General	1.86	1.12–3.08	
	Yes	0.212	0.03–1.44	
**APGAR 3rd minute**				
**Type of anaesthesia**	Regional	1	–	0.025
	General	2.33	1.11–4.86	
**APGAR at 5th minute**				
**Type of anaesthesia**	Regional	1	–	0.124
	General	2.81	0.75–10.53	
**Resuscitation**				
**Type of anaesthesia**	Regional	1	–	0.041
	General	1.90	1.02–3.51	
**Neonatal Asphyxia**				
**Type of anaesthesia**	Regional	1	–	0.765
	General	1.08	0.63–1.88	

## Discussions

The aim of our study was to assess the effects of regional versus general anaesthesia on foetal and maternal outcomes following caesarean section for acute foetal distress, to determine the safest anaesthetic technic to use in the case of this emergency. We also sought to analyse the influence of other maternal and foetal factors on foetal and maternal outcomes. We found that unlike SA, GA was associated with lower APGAR scores in the 1st 3rd and 5th minutes following CS. However, this finding was significant for the 1st and 3rd minutes only.

Our findings are consistent with those of several other studies in Africa, Europe, and the USA, where GA was seen to be associated with lower APGAR scores, following anaesthesia for AFD. However, unlike our study, their results showed that lower APGAR scores following GA was significant even at the 5th minute following delivery [12, 15, 16]. GA is shown in our study to be associated with significantly higher chances of neonatal resuscitation when compared to SA. We therefore might have thought that neonatal admission and morbidity would have consequently been increased. However, this was not the case in our study as expected. Thangaswamy et al. showed that in Category 1 CS, GA was significantly associated with greater frequency of neonatal unit admissions and mortality [15]. Several studies have shown that neonatal resuscitated babies have higher morbidity and sequalae of neonatal asphyxia [17, 18]. Failure of our study to tie with these findings may infer better resuscitative techniques in the context of DGH for babies born by caesarean section.

Confounders studied in the current study included previous CS, foetal heart rate pattern on CTG, and parturients referral from a different hospital. None of these were found to influence the choice of anaesthetic technique. We noticed that, whether patients were referred or not, and no matter the degree of heart rate abnormality, or whether they had had a past caesarean section, foetal outcome were still better if SA and not GA was used for CS. This finding is consistent with previously reported studies [12, 15]. Our study also went further to examine the association of other exposures such as gestational age, birth weight, gravidity, the use of ephedrine and fever on neonatal outcomes. Maternal fever was consistently shown to be associated be associated with lower APGAR scores, significantly so, in the first- and third-minutes following delivery. These results are not surprising and have been confirmed by other studies showing the impact of maternal fever on neonatal outcome [19–21]. Mothers presenting with fever should therefore be aggressively resuscitated in the perinatal wards prior to delivery to reduce the risks of poor foetal outcomes.

Maternal outcomes in our study surprisingly show that, women presenting with AFD who were operated, were likely to have a more favourable outcome if GA instead of SA was the technique of anaesthesia used. This is contrary to findings of other studies. Where regional anaesthesia is found to be associated with better maternal outcomes [22]. Thangaswamy et al. revealed that ICU admission and maternal mortality were also comparable between GA and SA [15]. However further studies will be needed to conclude on these observations.

The present study should also be interpreted in the context of its limitations. Firstly, we were not able to assess some factors such as the interval between the induction of anaesthesia and delivery of the neonate as this is a paramount factor influencing neonatal outcome with respect to anaesthetic technique. Secondly, maternal factors such as blood loss and postoperative pain were not studied. This was due to the retrospective nature of our study as this data was very inconsistently written in the medical records. However, using robust methods, we have contributed to the scarcity of data on the safest anaesthesia technique for CS indicated for AFD. These findings should contribute to informing decision making by anaesthesiologists, who are often confronted with the dilemma of which technique of anaesthesia to choose in this emergency. It has been a common clinical anaesthesia practice to perform GA in this situation. The current study shows that SA is safer than the traditional GA often used.

## Conclusion

The study infers associations between GA for CS indicated for AFD and neonatal morbidity on one hand and between SA and maternal morbidity and mortality on the other hand. This, however failed to translate into a difference in perinatal mortality. These results do not discount the role of GA in low-income settings, but we emphasise the need for specific precautions like close monitoring of labour for AFD and adequate anticipation of neonatal resuscitation when GA is used for CS in the case of AFD. Proper anticipation of maternal morbidity and mortality following SA is also warranted.

## Data Availability

The dataset analysed in this study are not publicly available because they are currently being analysed for a second research article. Nonetheless, they are available from the corresponding author on reasonable request.

## Abbreviations

AFD: Acute foetal distress
CTG: Cardiotocography
CS: Caesarean section
GA: General Anaesthesia
SA: Spinal Anaesthesia
LICs: Low-income countries
RATs: Regional anaesthesia techniques
NNA: Neonatal asphyxia
DGH: Douala General Hospital
PDPH: Post-dural puncture headache
ASA: American Society of Anaesthesia
FHR: Foetal Heart Rate
CRR: Crude risk ratios
CI: Confidence Interval
IQR: Interquartile Range
PPH: Post-partum haemorrhage
